# NK Cells and Other Innate Lymphoid Cells in Hematopoietic Stem Cell Transplantation

**DOI:** 10.3389/fimmu.2016.00188

**Published:** 2016-05-18

**Authors:** Paola Vacca, Elisa Montaldo, Daniele Croxatto, Francesca Moretta, Alice Bertaina, Chiara Vitale, Franco Locatelli, Maria Cristina Mingari, Lorenzo Moretta

**Affiliations:** ^1^Department of Experimental Medicine, University of Genova, Genova, Italy; ^2^Division of Regenerative Medicine, Stem Cells and Gene Therapy, San Raffaele Telethon Institute for Gene Therapy, IRCCS San Raffaele Scientific Institute, Milano, Italy; ^3^Department of Internal Medicine, University of Verona, Verona, Italy; ^4^Ospedale Sacro Cuore Negrar, Verona, Italy; ^5^Department of Pediatric Hematology and Oncology, IRCCS Bambino Gesù Children’s Hospital, Rome, Italy; ^6^U.O. Immunology IRCCS AOU San Martino-IST, Genova, Italy; ^7^Department of Pediatrics, University of Pavia, Pavia, Italy; ^8^Centro di Eccellenza per la Ricerca Biomedica – CEBR, Genova, Italy; ^9^Department of Immunology, IRCCS Bambino Gesù Children’s Hospital, Rome, Italy

**Keywords:** hematopoietic stem cell transplantation, innate lymphoid cells, NK cells, GVHD

## Abstract

Natural killer (NK) cells play a major role in the T-cell depleted haploidentical hematopoietic stem cell transplantation (haplo-HSCT) to cure high-risk leukemias. NK cells belong to the expanding family of innate lymphoid cells (ILCs). At variance with NK cells, the other ILC populations (ILC1/2/3) are non-cytolytic, while they secrete different patterns of cytokines. ILCs provide host defenses against viruses, bacteria, and parasites, drive lymphoid organogenesis, and contribute to tissue remodeling. In haplo-HSCT patients, the extensive T-cell depletion is required to prevent graft-versus-host disease (GvHD) but increases risks of developing a wide range of life-threatening infections. However, these patients may rely on innate defenses that are reconstituted more rapidly than the adaptive ones. In this context, ILCs may represent important players in the early phases following transplantation. They may contribute to tissue homeostasis/remodeling and lymphoid tissue reconstitution. While the reconstitution of NK cell repertoire and its role in haplo-HSCT have been largely investigated, little information is available on ILCs. Of note, CD34^+^ cells isolated from different sources of HSC may differentiate *in vitro* toward various ILC subsets. Moreover, cytokines released from leukemia blasts (e.g., IL-1β) may alter the proportions of NK cells and ILC3, suggesting the possibility that leukemia may skew the ILC repertoire. Further studies are required to define the timing of ILC development and their potential protective role after HSCT.

## Introduction

Allogeneic hematopoietic stem cell transplantation (allo-HSCT) still represents a major therapeutic option for severe hematological and immunological disorders (1). However, success of allo-HSCT can be impaired by graft-versus-host disease (GvHD) and, in the case of high-risk hematological malignancies, also by disease relapse. The clinical outcome can also be hampered by infections favored by the delayed immune reconstitution in transplanted patients (1). Moreover, only 60% of patients may find a related or unrelated HLA-matched donor. For the remaining patients, umbilical cord blood (UCB) may represent an alternative source of HSC (2). UCB transplant allows a less stringent HLA-matching between donor and recipient, but it is frequently associated with delayed neutrophil engraftment and delayed T-cell reconstitution. Moreover, UCB transplant recipient cannot benefit from the adoptive transfer of antigen-experienced donor T-cells (2). Another important therapeutic option is represented by the haploidentical (haplo)-HSCT. In this setting, the donor (in most instances, a parent) is identical for one HLA haplotype and mismatched for the other one (3). Given the high degree of HLA disparity, haplo-HSCT requires an extensive T-cell depletion of the graft (3) or heavily posttransplantation immune-suppressive therapy to prevent severe GvHD (4). In both UCB-HSCT and haplo-HSCT settings, the immune-compromised hosts are highly susceptible to a wide range of opportunistic infections. Thus, cells of the innate immunity are the only players exerting a major defensive role for several months before the restoring of adaptive immune responses. In particular, natural killer (NK) cells can provide protection against viral reactivation and/or primary infections. Perhaps, more importantly, the presence of alloreactive NK cells provides a potent graft-versus-leukemia (GvL) effect that contributes to tumor eradication (4, 5). It is now clear that NK cells are one of the components of a broad family of innate lymphoid cells (ILCs). However, so far, little is known on the possible role of the other ILC subsets in haplo-HSCT. Here, we will summarize our current knowledge on ILCs both in murine models and in human studies, since they could result crucial in host defenses after HSCT.

### ILC Subsets

Different from T-cells and B-cells, ILCs are a group of lymphocytes that do not express rearranged antigen-specific receptors (6). ILCs represent a heterogeneous family of cells classified on the basis of their transcriptional and functional profile. Similar to T-cells, ILCs have been grouped into cytotoxic-ILC and helper-ILC (6). NK cells represent the cytotoxic-ILC population (7). They express eomesodermin (Eomes) and T-box transcription factor (T-bet), display cytolytic activity, and produce pro-inflammatory cytokines, primarily IFNγ and TNF. Helper-ILC population is further subdivided into three groups, namely: ILC1, ILC2, and ILC3 (6). ILC1 depend on the expression of the T-bet transcription factor for their development and secrete IFNγ, but, different from NK cells, they neither express Eomes nor exert cytolytic activity (7). ILC2 express GATA-binding protein 3 (GATA3) and produce type-2 cytokines, including IL-13 and IL-5 (8). Finally, ILC3 are a heterogeneous cell population, including fetal lymphoid tissue-inducer (LTi) cells and adult ILC3 that are further subdivided into natural cytotoxicity receptors^−^ (NCR^−^) and NCR^+^ subsets. Collectively, ILC3 are defined by the expression of the retinoic acid receptor-related orphan receptor (RORγt) and produce mainly IL-17 and IL-22 (9). Studies in mice revealed that ILC, similar to T-cells and B-cells, derive from the common lymphoid progenitors (CLPs). The expression of the Id2 transcription factor determines further commitment toward a precursor common to all ILC subsets. While the NK cell precursor diverges early from the other ILC lineages, all helper-ILCs share a common helper-ILC progenitor (CHILP). Subsequently, upon exposure to different cytokines and/or to environmental cues, the CHILP differentiate toward ILC1, ILC2, or ILC3 (9). In humans, the developmental pathways are less characterized (10). However, NK and ILC3-committed precursors have recently been identified. Indeed, Renoux and coworkers identified, in several fetal and adult tissues, Lin^−^CD34^+^CD38^+^CD123^−^CD45RA^+^CD7^+^CD10^+^CD127^−^ cells able to differentiate exclusively toward cytotoxic NK cells both *in vitro* and *in vivo* (11). The ILC3 precursors, identified according to the Lin^−^CD34^+^RORγt^+^ phenotype, have been detected selectively in tonsils and intestinal lamina propria (12).

### ILC in Host Defenses against Pathogens and in Tissue Remodeling

**Figure 1:**
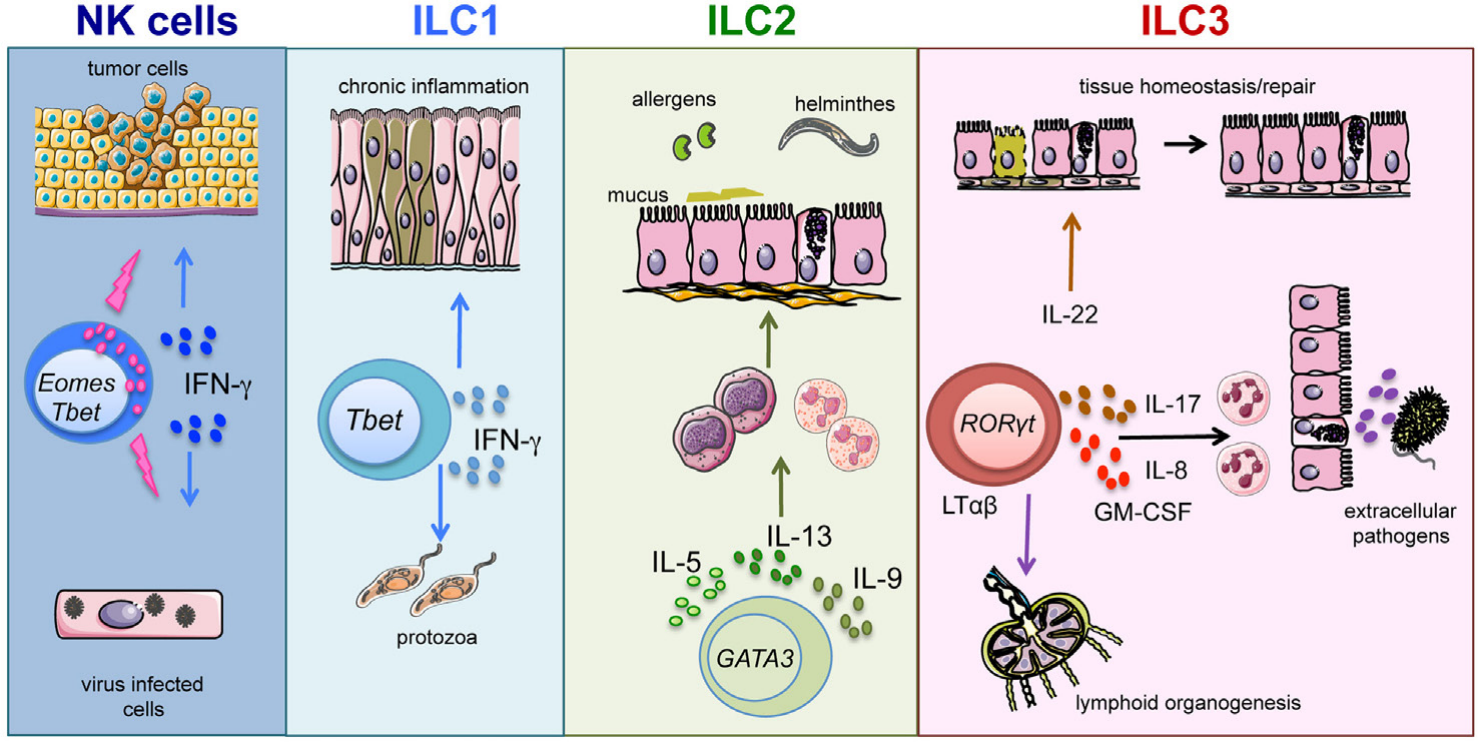
**ILC subsets and function**. Graphic representation of the role played by ILC subsets in host defenses and tissue homeostasis/repair.

In view of the heterogeneous cytokine profile and function of different ILC subsets, it is conceivable that ILCs may contribute to host defenses against a broad variety of pathogens (13, 14). Our knowledge on human ILC1 and their functional profile are still rather limited (15–17). Taking advantage of murine models, it has been shown that ILC1, thanks to the production of IFN-γ and TNF, contribute to immune responses against intracellular pathogens, such as *Toxoplasma gondii* (18). Also, NK cells are an important source of IFN-γ and TNF and, in addition, display very important effector functions, such as natural cytotoxicity and antibody-dependent cell-mediated cytotoxicity (ADCC). In the context of antimicrobial defenses, NK cells are primarily involved in the control of different viral infections, primarily herpes-viruses, but may also exert a protective role against bacterial and parasitic infections (19, 20). Of note, NK cells, thanks to their potent cytolytic activity, play also an important role against tumors (Figure 1) (21).

Host protection against parasites requires type-2 responses. A number of findings indicate that, during helminthic infections, epithelial cell-derived IL-25, IL-33, and thymic stromal lymphopoietin (TSLP) induce IL-13 release from ILC2 (22–24). In turn, IL-13 increases mucus production and smooth muscle contractility, thus contributing to the control of parasitic infections (25). In addition, dendritic cell (DC)-derived IL-23 and IL-1β cytokines induce the release of IL-22 and IL-17 by ILC3 (26–29). In turn, these ILC3-derived cytokines promote the production of antimicrobial molecules and neutrophil recruitment, enhancing the response against extracellular bacteria and fungi (14, 27, 30, 31) (Figure 1). In humans, the role played by ILC2 and ILC3 in defenses against pathogens is still undefined (10). Notably, patients experiencing helminthic infections show increases in ILC2 proportions; however, the role of ILC2 in anti-parasitic responses needs further investigation to be precisely clarified (8). Besides their antimicrobial function, ILCs are also involved in processes of tissues remodeling/repair. In particular, ILC2 appear to be involved in resolution of damages caused by viral or parasitic infections in lung tissues. Indeed, in response to IL-33, ILC2 also produce amphiregulin that promotes airway epithelial cell repair (32). Fetal LTi cells were the first ILC3 population to be described. LTi cells coordinate lymphoid organogenesis through their interaction with stromal cells by means of the LTαβ/LTβ receptor, leading to the upregulation of ICAM-1 and VCAM adhesion molecules on stromal cells (33). More recently, postnatal ILC3 have been shown to promote both survival and proliferation of stromal cells, following lymphoid tissue damage caused by viral infection and/or irradiation (34) (Figure 1). In addition, ILC3-derived IL-22 exerts a protective role on intestinal epithelial stem cells, particularly in the context of tissue damage caused by irradiation and/or acute GvHD (35, 36).

### ILC and HSCT

So far, only a limited number of studies addressed the role of helper-ILC in the context of HSCT (35–37).

#### Reconstitution

Chemotherapy and radiotherapy treatment before HSCT induces extensive tissue damages in the host, including severe intestinal mucositis (38). Such damages can be even worse after allo-HSCT, if donor T lymphocytes attack the recipient intestinal epithelium (GvH reaction) (39). In a murine model of acute GvHD, Hanash and coworkers showed that host-derived IL-22 could substantially limit the development of GvHD (35). They could identify intestinal ILC3 subset as a main producers of IL-22 after total body irradiation treatment. In particular, IL-22 seemed to play a crucial role in the protection against epithelial cell damage and in preserving intestinal stem cells. These data are further supported by the finding that treatment with IL-22, in mice receiving bone marrow transplantation, resulted in increased intestinal stem cell recovery, in enhanced epithelial cell regeneration, and in reduction of intestinal GvHD (36). Given the role of ILC3 in lymphoid organogenesis and in lymphoid tissue remodeling, a role for these cells could also be envisaged in the regeneration of lymphoid tissues damaged by radiations (38, 40). Of note, ILC3-derived IL-22 can also favor the recovery of thymic epithelial cells, thus allowing a more efficient and rapid reconstitution of T-cell compartment (Table 1) (41). Conversely, it remains to be determined whether ILC3 also contribute to the regeneration of secondary lymphoid organs. In this context, it is recently shown that gamma irradiation used in conditioning regimen before HSCT may exert a long-lasting effect on secondary lymphoid organ structure and function (40). Also, ILC2 appear to be involved in epithelial tissue repair, particularly in lung tissues; however, no data are available to support an actual protection exerted by these cells in GvHD-induced tissue damages (42).

**Table 1:** **This table summarizes the main function exerted by distinct ILC subsets and the possible role exerted by these cells in the context of HSCT**.

Cell type	Function	Role in HSCT
NK cells	Anti-tumor activity (21)	GvL (21, 55, 56)
	Defense against virus-infected cells (19, 20, 53)	Control of viral reactivation and/or primary infections (55, 56)
ILC1	Defense against protozoa (14, 18)	*Control of posttransplant opportunistic infections?*
ILC2	Defense against helminthic infection (14, 22–24)	*Control of posttransplant opportunistic infections?*
	Wound healing (32)	*Contribute to tissue repair?*
ILC3	Lymphoid organogenesis (33)	*Regeneration of secondary lymphoid organs?*
	Lymphoid tissue remodeling (34)	Thymic epithelial cell recovery (41)
	Epithelial homeostasis (35, 36)	Protection against therapy-induced epithelial damage and mucositis and promotion of tissue regeneration (35, 37)
		Reduction of GvHD occurrence (37)
	Defense against extracellular bacteria and fungi (14, 27–30)	*Control of posttransplant opportunistic infections?*

*Only for some of the ILC populations, a role in the context of HSCT has been demonstrated. The possible roles exerted by other cell subsets are indicated in italics*.

#### Graft-versus-Host Disease

In the context of human HSCT, only a single study investigated the possible role of ILCs in the protection from GvHD. It was suggested that both host and donor ILCs might exert a protective role (37). The expression of activation markers and of gut and skin homing receptors on host ILCs, detected prior to HSCT, correlated with a lower incidence of both mucositis and GvHD. Notably, after HSCT, ILCs detectable in peripheral blood (PB) are of donor origin. An early appearance of activated NCR^+^ILC3 correlated with reduced risk of developing GvHD. In light of these finding, it is conceivable that the induction of a rapid ILC3 expansion/generation after HSCT may protect from GvHD. In this context, we have recently shown that granulocyte-colony-stimulating factor (G-CSF) could affect ILC3 and NK cell differentiation (43). Of note, G-CSF is used in UCB transplantation to accelerate engraftment and neutrophil recovery and is also used as a potent HSC mobilizing agent, before collection of HSC from donor PB (2, 44). Accordingly, we observed that HSC recovered after G-CSF-induced mobilization display a delayed and lower ILC3 and NK cell differentiation *in vitro* as compared to HSC isolated from bone marrow or UCB (43). These findings suggest that pre- and posttransplant treatment with G-CSF may affect ILC3 generation. Further studies should confirm these results *in vivo* and establish possible correlations with the occurrence of GvHD. Of note, it has been shown that ILC development may be impaired in patients with acute myeloid leukemia (AML) (45). Thus, after HSCT, ILC development might be affected by the presence of high residual leukemia burden or leukemia relapse. Indeed, it has been shown that HSC, when cultured in the presence of IL-1β-releasing AML blasts, display an impaired ability to differentiate toward ILC3 (46). Although in these culture setting the generation of NK cells seemed to be favored over ILC3, the final number of NK cells recovered was dramatically lower than those recovered in control cultures. Thus, if this inhibitory effect occurs also *in vivo*, it could have a negative impact on the NK-mediated GvL in haplo-HSCT. Of note, NK cell generation and differentiation after HSCT may be affected by immune-suppressor drugs, such as calcineurin inhibitors, used for treatment of GvHD (47, 48). On the other hand, helper-ILC reconstitution does not seem to be affected by cyclosporine or corticosteroids (37).

#### Opportunistic Infections

Studies in mice revealed that ILC might contribute to host defenses against different pathogens. In particular, while they are crucial in the control of infections in immune-compromised mice (18, 28, 49, 50), their actual role in the presence of a functional T-cell compartment seems to be marginal [as in the case of ILC3 during *Citrobacter rodentium* infection (51)]. However, as discussed above, patients transplanted with UCB cells or recipients of T-cell depleted haploidentical allograft experience a delayed recovery of both T-cell and B-cell adaptive responses, thus suggesting a possible relevant role of ILC in these transplantation settings. Accordingly, a rapid ILC differentiation after HSCT could guarantee an efficient host defense against opportunistic infections. Whether ILC1, ILC2, and ILC3 may indeed play a role in the control of infections in immune-compromised host, such as HSCT patients, has not been addressed yet. In contrast, clear evidence exists that patients with NK cell deficiencies and patients with functional NK cell defects display a higher susceptibility to viral infections [reviewed in Ref. (52)]. Moreover, in humanized mouse models, NK cells are required to effectively control Epstein–Barr virus (EBV) reactivation even in the presence of CD8^+^ T-cells (53). NK cells also contribute to host protection against cytomegalovirus (CMV) (20, 54) also in the context of HSCT (55). In particular, NK cell involvement in CMV control is suggested by the finding that certain KIR haplotypes correlate with decreased CMV reactivation after transplantation (56). On the other hand, CMV, similar to other viral infections, can dramatically shape the NK cell repertoire (57–68). In humans, CMV infection is accompanied by a rapid NK cell maturation, the acquisition of KIR and CD57, and a selective expansion of a NKG2C^+^ NK subset (62, 63, 67).

## Concluding Remarks

Information available on ILC development and function derives primarily from studies performed in mice. Although these studies could provide reliable models of ILC differentiation, further analyses are required to address the dynamics of helper-ILC reconstitution after HSCT, the influence of HSC source, and the possible interference of cytokines produced by leukemia cells with ILC development. In addition, it will be crucial to clarify the role of specific ILC subsets in response to infections. Key information is still lacking in humans, not only on the role of ILC during infections but also in lymphoid tissue homeostasis. The possible exploitation of ILC in the context of HSCT requires a deeper knowledge of the mechanisms regulating their function and of the stimuli that drive their development.

## Author Contributions

All the authors provided data reported in this review. PV, EM, CV, and LM wrote and revised the manuscript.

## Conflict of Interest Statement

The authors declare that the research was conducted in the absence of any commercial or financial relationships that could be construed as a potential conflict of interest.
